# Discovery of a Metastatic Immune Escape Mechanism Initiated by the Loss of Expression of the Tumour Biomarker Interleukin-33

**DOI:** 10.1038/srep30555

**Published:** 2016-09-13

**Authors:** Iryna Saranchova, Jeffrey Han, Hui Huang, Franz Fenninger, Kyung Bok Choi, Lonna Munro, Cheryl Pfeifer, Ian Welch, Alexander W. Wyatt, Ladan Fazli, Martin E. Gleave, Wilfred A. Jefferies

**Affiliations:** 1 The Michael Smith Laboratories, University of British Columbia, 301-2185 East Mall, Vancouver, BC Canada V6T 1Z4; 2 Department of Microbiology & Immunology, University of British Columbia, 1365 - 2350 Health Sciences Mall, Vancouver, BC Canada V6T 1Z3; 3 Department of Animal Care Services, University of British Columbia, 4145 Wesbrook Mall, Vancouver BC Canada V6T 1W5; 4 Department of Urologic Sciences, University of British Columbia Gordon & Leslie Diamond Health Care Centre, Level 6, 2775 Laurel Street, Vancouver, BC Canada V5Z 1M9; 5 The Vancouver Prostate Centre, University of British Columbia 2660 Oak Street, Vancouver, BC Canada V6H 3Z6; 6 Department of Medical Genetics, University of British Columbia 1364 - 2350 Health Sciences Mall, Vancouver, BC Canada V6T 1Z3; 7 Department of Zoology, University of British Columbia 4200 - 6270 University Blvd, Vancouver, BC Canada V6T 1Z4; 8 Centre for Blood Research at the University of British Columbia 4th Floor - 2350 Health Sciences Mall, Vancouver, BC Canada V6T 1Z3; 9 Djavad Mowafaghian Centre for Brain Health at the University of British Columbia 2215 Wesbrook Mall, Vancouver BC Canada V6T 1Z3.

## Abstract

A new paradigm for understanding immune-surveillance and immune escape in cancer is described here. Metastatic carcinomas express reduced levels of IL-33 and diminished levels of antigen processing machinery (APM), compared to syngeneic primary tumours. Complementation of IL-33 expression in metastatic tumours upregulates APM expression and functionality of major histocompatibility complex (MHC)-molecules, resulting in reduced tumour growth rates and a lower frequency of circulating tumour cells. Parallel studies in humans demonstrate that low tumour expression of IL-33 is an immune biomarker associated with recurrent prostate and kidney renal clear cell carcinomas. Thus, IL-33 has a significant role in cancer immune-surveillance against primary tumours, which is lost during the metastatic transition that actuates immune escape in cancer.

Cancer is the leading cause of death in the developed world^1^ and often arises as a result of complex symphony of somatic genetic mutations that occur in oncogenes and tumour suppressor genes. These mutations cause uncontrolled cell proliferation and often enable primary tumours to continue to mutate and evolve into the metastatic form of the disease that is often lethal^2^,^3^. Genomic profiling of tumours has revealed a “metastatic gene signature” common to many different tumour types^2^. This metastatic signature distinguishes tumours with localized growing potential from those genetically configured to disseminate to distant sites and includes genes mediating tumour cell motility, invasion, immune evasiveness, angiogenesis and colonization.

The immune system serves to limit the emergence of tumours. The cellular arm of the immune system is critical in providing protection against tumours. CD8^+^ T cells of the immune system are able to distinguish between normal cells and cancerous or virus-infected cells by monitoring major histocompatibility complex class I (MHC-I) molecules on the cell surface^4^,^5^. MHC-I molecules, which are displayed by virtually all cells, form a complex with fragments of protein, or peptides, from the cell. Tumour cell peptides related to the neoplastic transformation events may be presented by MHC-I molecules on the tumour cell surface, enhancing tumour-specific T cell reactivity.

The gene expression profile in tumour tissue is influenced, to a significant extent, by the local microenvironment through the connective tissue framework. This framework consists of normal cells, including stromal fibroblasts, infiltrating immune cells, as well as extracellular matrix. It is now generally accepted that tumour-infiltrating immune cells are intimately linked to the kinetics of tumour growth, via direct contact and via chemokine-, cytokine-related signalling pathways. IL-33 is a cytokine belonging to the IL-1 superfamily, which is produced in different organs (lungs, prostate, cervix, pancreas)^6^,^7^,^8^,^9^,^10^. IL-33 is a dual-function protein that acts as a nuclear factor and as a pro-inflammatory cytokine. Nuclear localization and association with heterochromatin is mediated by the N-terminal domain and allows IL-33 to function as a novel transcriptional regulator of the p65 subunit of the NF-κB complex. The C-terminal domain is sufficient for binding to the ST2 receptor and activating the production of type 2 cytokines (*e.g.* IL-5 and IL-13) from polarized T helper (Th2) cells^11^ and Type 2 innate lymphocytic cells (ILC2)^12^. IL-33 is important for the development and function of ILC2s^13^,^14^,^15^, and has been shown to have the direct effect on other immune cells including eosinophils, mast cells, basophils, natural (NK) killer cells, NK T cells, T regulatory cells (T reg), which constitutively express ST2 receptors in a GATA3/STAT5-dependent manner^16^,^17^,^18^,^19^,^20^,^21^,^22^,^23^. Most recently, CD4^+^ and CD8^+^ T cells were shown to transiently express ST2 receptor upon activation and to participate in IL-33-signalling^24^,^25^. The recent literature regarding IL-33 involvement into tumorigenesis is controversial, apparently showing both immunoprotective^26^,^27^ and tumour-promoting effects^27^,^28^,^29^,^30^,^31^,^32^,^33^, depending on the site of origin and clinical stage of the disease.

The present study seeks to unravel and explain these findings, while uncovering a hitherto undescribed mode of immune evasion in metastatic cancer. We demonstrate that the expression of IL-33 plays a protective role during the transition from primary to metastatic tumours. We find that down-modulation of IL-33 and MHC-I/HLA related genes are associated with progression to metastatic disease and discover that IL-33 downregulation is a biomarker associated with recurrence in human metastatic prostate and kidney renal clear cell carcinomas. Furthermore, we have found that IL-33 gene expression by tumours complements MHC-I production and immune recognition of processed tumour antigens, and, consequently, reduces the frequency of circulating tumour cells and also the growth of metastatic tumours *in vivo.* Overall, this study demonstrates that immunosubversion of the IL-33-dependent antigen processing pathways in metastatic forms of the cancer is a new paradigm for understanding both immune-surveillance and immune escape in malignancy.

## Results

### IL-33 expression is decreased in metastatic carcinomas

To address the metastatic gene signature network, we conducted a comparative microarray analysis on antecedent non-metastatic and metastatic cell lines of both murine lung and prostate cancers, using the Agilent Two Color Microarray Technology (a 28005 Two-Color Agilent microarray with a total of 55821 probes). Analysis of the aberrant expression of genes in the metastatic/non-metastatic cell lines provided an avenue to begin to characterize and understand the mechanism of transition from immunologically-recognized to immunoevasive tumours. To validate the mouse data, expression of selected genes within human tumours was taken into account, using the data obtained from the Gene Expression Atlas created by the European Biostatistics Institute (Gene Expression Atlas; https://www.ebi.ac.uk/gxa/home; access date: 30/01/2013). From these, we focused on genes known to be involved in novel aspects of inflammation or immunity and those that may interact with the antigen presentation machinery.

**Figure 1:**
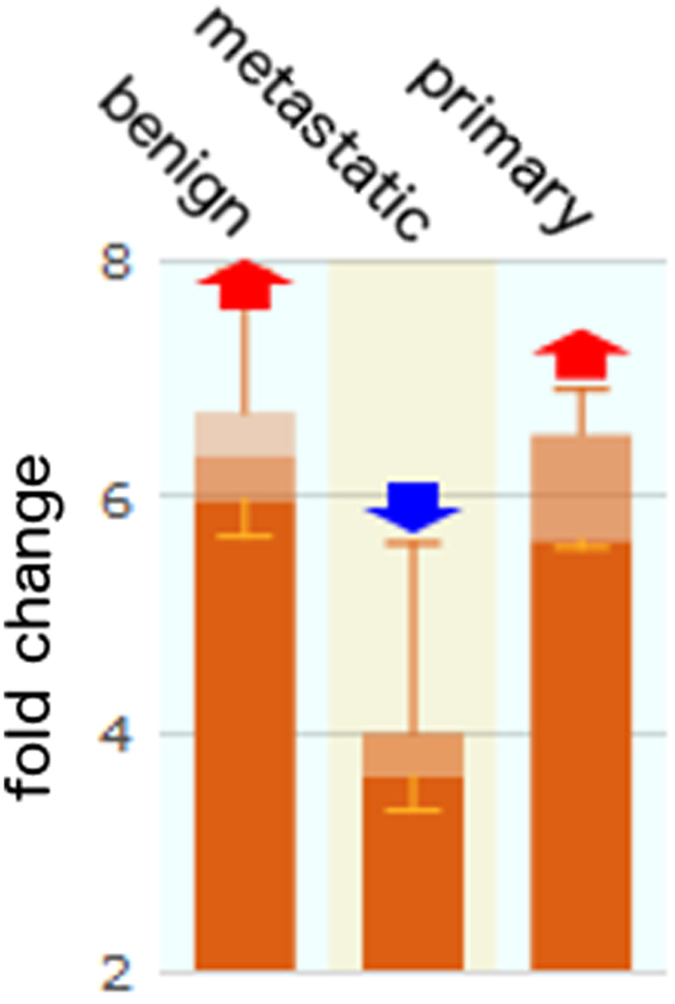
IL-33 expression is reduced in human metastatic prostate cancer. IL-33 gene is down-regulated in human metastatic prostate cancer compared to human benign and primary prostate tumors. The data was obtained from the Gene Expression Atlas created by the European Biostatistics Institute (Gene Expression Atlas; https://www.ebi.ac.uk/gxa/home; access date: 30/01/2013). A high-throughput immunoblot approach was utilized to characterize proteomic alterations in human prostate cancer progression, focusing on the transition from clinically localized prostate cancer to metastatic disease. They examined protein expression levels and gene transcript levels and found that the proteins that were in agreement with gene expression could be used as a predictor of clinical outcome.

While many genes were found to be downregulated in metastatic cancer as compared to primary cancer, one gene in particular, IL-33, was selected for further study since it is a key inducer of helper T cells and innate immune cells. According to our microarray data, IL-33 is down-regulated in the metastatic murine prostate and lung carcinomas (data not shown; unpublished). The IL-33 gene was confirmed to be down-regulated by 3–6 fold by separate qRT-PCR from two sets of primary and metastatic murine cell lines (Lung TC1/A9; Prostate PA/LMD) grown *in vitro*. The gene expression level in the data was in concordance with its protein expression level, indicating that it may be a good predictor for metastatic potential in carcinomas. In support of this hypothesis, it was also found to be significantly downregulated in metastatic human prostate carcinomas found in the Gene Expression Atlas (Fig. 1).

### MHC-I expression is tied to the level of IL-33

**Figure 2:**
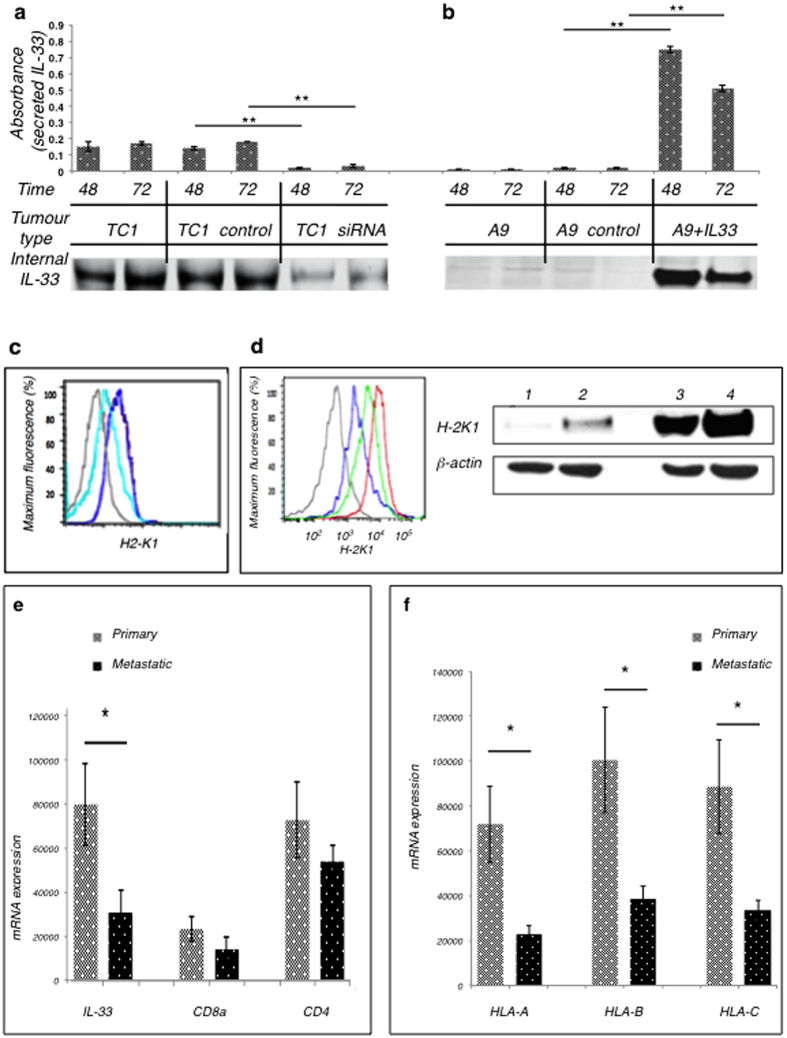
MHC-I expression is tied to the level of IL-33. **(a,b)** (top) Level of secreted IL-33 in supernatants measured by ELISA. **(bottom)** Level of IL-33 protein in the cell pellet measure by Western Blot analysis. **(a)** Down-regulation of IL-33 decreases secreted and intracellular IL-33 protein levels. Primary TC1 cells were treated with either scrambled siRNA or siRNA targeted against IL-33 for 48h or 72h. **P < 0.005 (Student’s t-test), comparing IL-33 secretion of TC1 cells transfected with siRNAs untargeted or targeted against IL-33. **(b)** Over-expression of IL-33 increases secreted and intracellular IL-33 protein levels. Metastatic A9 cells were transiently transfected with IL-33 gene or vector alone, and protein expression was tested after 48h and 72h. **P < 0.005 (Student’s t-test), comparing IL-33 secretion by A9 tumour cells transfected with IL-33 gene or vector alone. **(c)** Down-regulation of IL-33 gene corresponds with a decrease in MHC-I protein expression. Surface expression of MHC-I on TC1 cells was measured using flow cytometry, after cells were treated for 72h with siRNA: anti-IL-33 siRNA (**aqua**), untargeted siRNA (**blue**), no stain control (**grey**). **(d)** Over-expression of IL-33 gene increases MHC-I protein in tumour cells. Expression of MHC-I on A9 tumour cells was measured 48 h after transfection with IL-33. **(d, left)** Flow cytometry: An increase in IL-33 expression resulted in increased MHC-I surface expression on A9 cells: A9 untransfected control (**grey**); A9+vector alone (**blue**); A9+IL-33 (**green**); A9 treated with IFN-gamma, positive control (**red**). **(d, right)** WesternBlot: Increase in IL-33 gene expression restores A9 MHC-I protein expression. Lane 1 = A9 cells, Lane 2 = A9+vector; Lane 3 = A9+IL-33; Lane 4 = A9+IFN-gamma cytokine (50ng/ml). ß-actin was used as loading control. **(e,f)** HLA expression is co-regulated with IL-33 levels in human prostate cancer. Expression levels of mRNA of IL-33, CD8a, CD4 **(e)** and HLA-A, HLA-B, HLA-C **(f)** in castration-resistant prostate cancer (CRPC) are low relative to benign prostate tissue and both low- and high-risk primary tumours. D’Amico Risk classification: low-risk = prostate-specific antigen (PSA) ≤ 10, Gleason score ≤ 6, and clinical stage T1-2a; high-risk = PSA > 20, Gleason score ≥ 8, or clinical stage T2c-3a. *P < 0.05 (Student’s t-test) when compared high-risk primary (treated) tumours and metastatic CRPC.

To address the role of IL-33 on the expression of MHC-I within the tumour, IL-33 gene expression was inhibited using siRNA on primary tumour cells (TC1). After siRNA treatment for 72 to 96 h, both the IL-33 and MHC-I surface expression levels were assessed. The down-regulation of IL-33 protein was confirmed by ELISA (for secreted IL-33) and Western blot analysis (for cell-associated IL-33) (Fig. 2a). Primary lung carcinoma cells, TC1, treated with IL-33-directed siRNAs had a much lower abundance of MHC-I complexes than did controls (Fig. 2c), whereas the overexpression of IL-33 in metastatic cells (A9) (Fig. 2b) had a higher level of MHC-I production compared to untransfected controls (Fig. 2d). This suggested that IL-33 may be important for the expression of the immune recognizable phenotype of the primary TC1 cell tumour.

To evaluate the correlation between IL-33 and MHC/HLA expression (and consequent T cell infiltration) in human tumors, we used RNA-sequencing data collected from resected prostate tumours. In collaboration with Vancouver Prostate Centre, we assessed the expression of Human leukocyte antigen (HLA) and IL-33 in primary treated (high risk neo-hormone-treated (NHT)) and metastatic treated castration-resistant prostate cancer (CRPC) samples. We found that HLA-A, HLA-B, HLA-C genes have a similar pattern of reduction in CRPC and elevation in high risk NHT primary tumours (Fig. 2e,f). This also appears to parallel the IL-33 pattern and is a trend for TAP1, CD4 and CD8 genes as well. These data also suggested that IL-33 might be important for the expression of an immune-recognizable phenotype of primary tumours. In human prostate cancer, MHC-I/HLA and IL-33 are co-ordinately down-regulated during the metastatic re-programming of primary tumours revealing the involvement of the IL-33 gene in tumorigenesis and immune-escape.

### IL-33 expression reverses the lack of MHC-I, antigen processing and immune recognition of metastatic tumours

**Figure 3:**
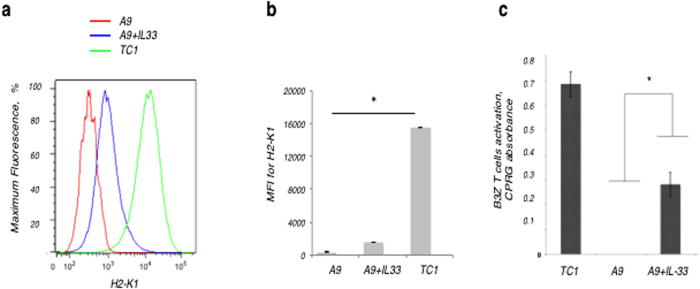
IL-33 gene restores MHC-I expression and signaling in MHC-I-loss metastatic carcinoma cells, as well as immune recognition of tumours. (**a,b**) MHC-I surface expression correlates with increased IL-33 expression. Mean Fluorescence Intensity (MFI) of MHC-I for stably transfected clones: metastatic A9, A9+IL-33, primary TC1; P < 0.05 when compared TC1 cells with A9 cells (Student’s t-test). **(c)** Functional B3Z-assay: the expression of IL-33 enhanced the ability of the tumour cells to present the **MHC-I** OVA-peptide complexes on their surface to be specifically recognized by B3Z T-cells. Values were normalized to vector alone control; *P < 0.05 compared with IL-33 expressing A9 cells (Student’s t-test).

Down-regulation of the MHC-I expression has been shown to be a primary indicator for rapid tumour progression and metastases in human prostate cancer^34^,^35^,^36^. Moreover, a very recent large-scale genomic study of 18 types of solid human tumour biopsies (including: uterine, breast, colorectal, stomach, kidney, lung, prostate and other cancers) has highlighted loss of antigen presentation with mutations in beta-2-microglobulin, HLA-A, HLA-B and HLA-C, supports our earlier findings in lung carcinoma^37^,^38^,^39^, as a key strategy in tumour immune evasiveness^40^. Therefore, we next investigate the effects of IL-33 on MHC-I expression and functionality. The pIRES2-EGFP-expressing vector system (both plus and minus the IL-33 gene) was stably transfected into TC1 and A9 cells, matched antecedent primary and metastatic cell lines. The EGFP was constitutively expressed and allowed green GFP-expressing tumour cells to be sorted and stable clones to be isolated. Whereas primary TC1 cells are known to express TAP-1 and MHC-I, metastatic A9 cells have greatly reduced expression of these genes^41^. Overexpression of IL-33 in transfected A9 cells induced increased expression of MHC-I (Fig. 3a,b), linking IL-33 to antigen presentation and suggesting that IL-33 acts upstream of this pathway to restore MHC-I expression in tumour cells.

To evaluate the functionality of MHC-I induced by IL-33, the ability of stably transfected tumour cells to present an MHC-I - restricted epitope complex was assessed using a B3Z T cell assay. The B3Z T cell line is a lacZ-inducible CD8^+^ T cell hybridoma genetically modified to show the activation through the only one signal - recognition of MHC-I in association with the specific antigen from ovalbumin (OVA 257−264; SIINFEKL)^42^. After incubation with soluble SIINFEKL, the transfected tumour cells were cultured with B3Z T cells. An increased activation of the B3Z T cells corresponds to a higher number of MHC-I:OVA complexes presented on the tumour cell surface. A9 cells stably transfected with IL-33 gene were able to present a higher number of MHC-I:SIINFEKL complexes on their surface than did A9 cells transfected with vector alone (Fig. 3c). This result showed that IL-33–expressing A9 cells had a greater capacity for B3Z T cell priming and activation. These data directly demonstrate that IL-33 expression improves immune recognition of the murine metastatic lung carcinoma *in vitro*.

### IL-33 gene-complementation reverses metastasis *in vivo*

To test whether the IL-33-induced increase in MHC-I expression in the metastatic A9 cell line could contribute to the anti-tumoural effect of *IL-33*-gene *in vivo*, a mouse study was conducted. Tumour cells were initially injected subcutaneously into the right flank and local tumour growth was monitored. Use of the stably transfected A9 and TC1 cells, which constitutively expressed EGFP, allowed the green tumour cells to be tracked for spread beyond the initial site of injection.

**Figure 4:**
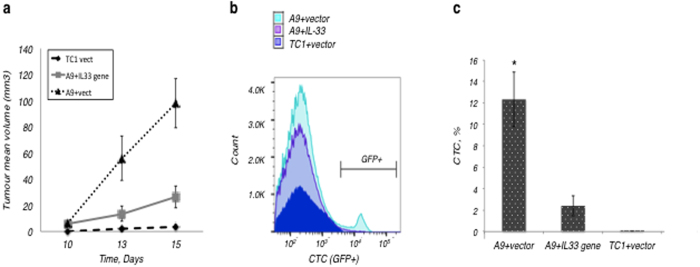
*IL-33* gene-complementation suppresses tumour growth rate *in vivo* and inhibits metastatic spread of tumour cells in a mouse model. (**a**) Stable transfection of *IL-33* gene into A9 cells resulted in significantly inhibited tumour formation in mice; P < 0.05, when comparing IL-33 expressing tumours (TC-1 or A9+IL-33) to A9 alone (Student’s t-test). (**b**) GFP-positive circulating tumour cells were detected in adrenal glands that were distal from initial subcutaneous inoculation, and assessed using flow cytometry. (**c**) Quantification of GFP-positive circulating tumour cells. The percentage of all cells was calculated from the fraction of live cells in 5 × 10^5^ events used to create a profile for each organ. The graph corresponds to the data from eight representative animals. *P < 0.05, comparing GFP-positive CTCs isolated from the primary (TC1) and metastatic (A9) bearing animals (Student’s t-test).

We found that expression of IL-33 significantly inhibited metastatic A9 tumour formation. The mean volume of tumours grown from A9+IL-33 cells was ~30–45% lower than from A9 controls, although both were higher as compared to the mean volume of primary TC1 tumours over the duration of study (Fig. 4a). Moreover, we observed that mice injected with A9 alone also suffered from severe clinical presentation in the form of significant weight loss and tumour ulceration (data not shown), which partially determined the length of the tumour trial. Animals with TC1 (primary tumours) and A9+IL-33 tumours better maintained or gained weight throughout the study. Tumour ulcerations (stage 4–5) with bleeding of adjacent tissues were detected for some cases in the group injected with metastatic A9 cells, but this was not seen in animals injected with primary TC1 or metastatic A9+IL-33 tumours.

The most important and unfavourable prognostic factor for the clinical course of tumour development is the metastatic spread of the disease. Therefore, the appearance of circulating tumour cells (CTCs) in disaggregated tissues was assessed in the most common metastatic sites for lung carcinoma: brain, lungs, liver, adrenal glands, lymph nodes and blood tissue. The highest percentage of GFP-positive CTCs were detected in liver (up to 32.0%) and adrenal glands (up to 16.0%) of animals bearing A9 tumours. IL-33 expression by the A9+IL-33 tumour cells reduced the average number of GFP-positive cells to ~0.15% in liver, and to ~2.63% in the adrenal glands (Fig. 4b,c; Table 1). Only single tumour cells were detected from lung and blood, while all the brain samples appeared tumour-free. CTCs were not detected from any of the tested tissues and organs of animals bearing primary tumours (TC1) with local growing potential, which are not genetically programmed to disseminate to distant sites (Table 1). Collectively, these observations suggest that *IL-33* gene-complementation can modify the malignant gene expression programming. As a result, IL-33 expression decreases the metastatic potential of the cancer cell population reducing the *in vivo* tumour growth rate, metastatic spread of the disease and its severity.

### Tumour-infiltrating immune cells

**Figure 5:**
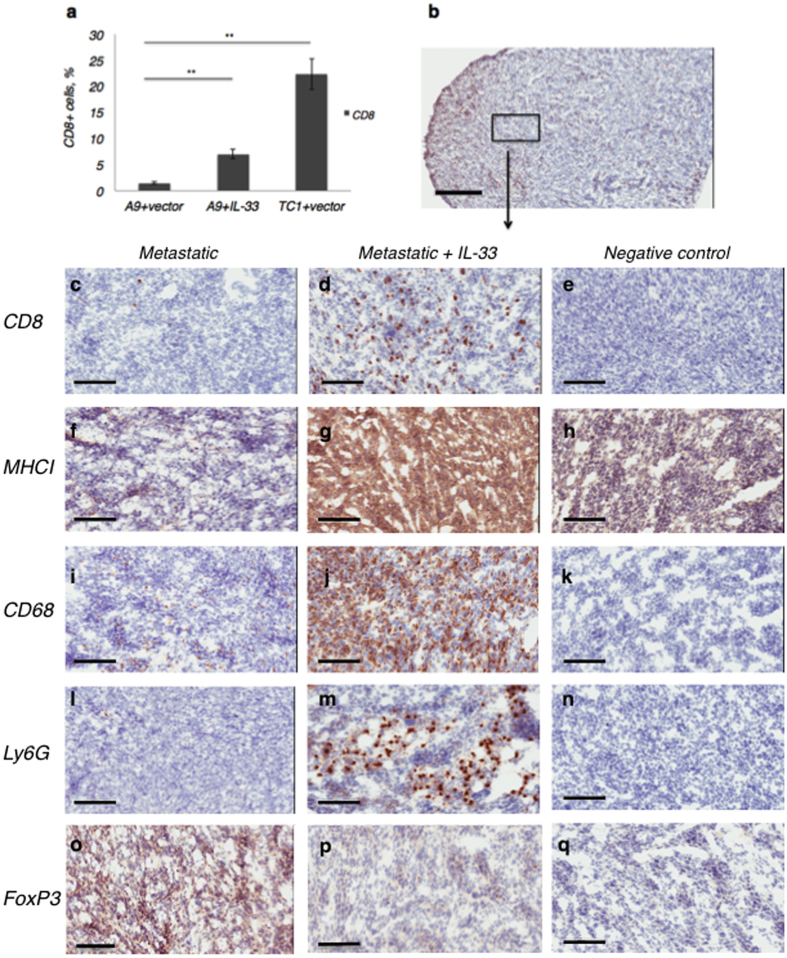
Genetic complementation of immune evasive tumours shows clear phenotypic shift towards immune recognition. **(a)** To overcome the issue of multi-focality, FACS analysis was used to count tumour-infiltrating lymphocytes (TILs). The expression of IL-33 by the tumour skews the TILs towards a cytotoxic T cell response, as a statistically significant increase in the number of CD8-positive cells can be shown in genetically modified (A9+IL-33) tumours versus in metastatic (A9), (Student’s t-test), **P < 0.005. **(b–q)** Immunohistochemical staining was used to support the FACS analysis. CD8 is primarily a marker for cytotoxic T cells, but also found on natural killer cells and dendritic cells; MHC-I is found on all nucleated cells; CD68 is a marker of monocytes and macrophages; Ly6G is a marker for monocytes, granulocytes and neutrophils; FoxP3 is a marker of regulatory T cells. Greater numbers of CD8-positive cytotoxic T cells can be seen within the genetically modified (A9+IL-33) tumours (**b**,**d**) versus unmodified metastatic (A9) tumours (**c**). IL-33 expression increases MHC-1 expression on tumour cell surface; A9+IL-33 **(g)** versus unmodified metastatic A9 **(f)**, thereby increasing tumour antigen presentation. Fewer regulatory T cells are present in IL-33 expressing tumours, as indicated by lower FoxP3 staining; metastatic A9 **(o)** versus A9+IL-33 **(p).** Increased macrophage and neutrophil response is seen in IL-33 expressing tumours; unmodified Metastatic A9 **(i**,**l)** versus A9+IL-33 **(j,m)** respectively. **(e**,**h**,**k**,**n**,**q)** Negative controls (rat IgG targeting Keyhole Limpet Hemocyanin (KLH)) were included to show that non-specific staining was minimized. 10 μm thick sections were stained with appropriate antibodies and imaged at 5x **(b)** or 20x **(c–q)** magnification. Size bar = 400 μm in **(b)**; Size bar = 100 μm in **(c–q)**.

**Figure 6:**
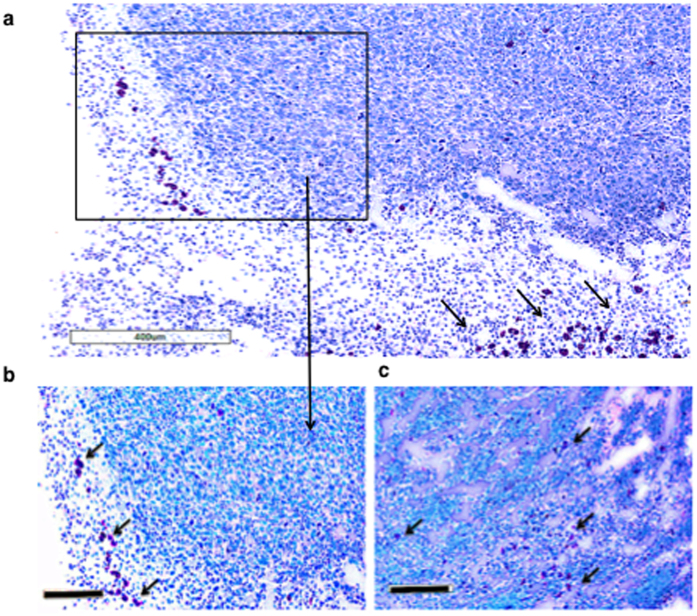
Eosinophil recruitment into tumour tissue is induced by IL-33 expression (C57BL/6 mouse). (**a,b)** Eosinophils are located on the border between metastatic (A9) tumour and normal tissue. **(c)** IL-33-induced changes allow eosinophilic infiltration into tumour tissue, which expresses IL-33. 10 μm thick tumour sections were stained with Giemsa stain and imaged at 5x (Size bar = 400 μm (**a**)) and 10x magnification (Size bar = 200 μm (**b,c**)).

Tumour-infiltrating immune cells are intimately linked to the kinetics of tumour growth^43^,^44^. The involvement of innate immunity (*e.g.* ILC2s, neutrophils, macrophages, eosinophils) and adaptive immunity (CD4^+^ and CD8^+^ T cells) were assessed by flow cytometry and immunohistochemistry. Up-regulated immune recognition shown previously by cytotoxic CD8^+^ T lymphocytes (CTLs) in the B3Z T cell assay (Fig. 3c) was well in line with the number of CD8^+^ tumour-infiltrating lymphocytes (TIL) detected in resected tumours by FACS analysis and seen within tumour sections (Fig. 5). In disaggregated tumour tissues, FACS analysis indicated CD8^+^ T cell content was lower in metastatic A9 tumours than in either the primary (TC1) or the modified metastatic A9+IL-33 tumours (Fig. 5a). This data was supported by immunohistochemical visualization of tumour tissue sections, which showed increased staining intensity for MHC-I-positive cells within IL-33-expressing tumours when compared to metastatic untreated tumours (Fig. 5b–h). Moreover, the number of CD8+ T cells was indirectly related to the number of circulating tumour cells. The percentage of circulating tumour cells (CTC) that could be detected in distal organs was significantly lower in IL-33-expressing tumours. Reduced CTCs also corresponded to increased percentages of CD8+ cytotoxic T ccells found in both the tumour and in local lymph nodes, suggesting that IL-33-induced changes may provide favorable conditions for the immune system to reduce metastatic spread, possibly by means of CD8+ T cells (Table S1). Interestingly, the metastatic A9 tumour microenvironment stained intensely for suppressive T-regulatory cell marker (FoxP3^+^), while this was not seen in the A9+IL-33 expressing tumour environment or in the negative control (Fig. 5o–q) that are also associated with tumour immune-resistance. However, the expression of IL-33 by the tumours appeared to prevent the accumulation of these immune-suppressive cells, and promoted intensive infiltration by CD68^+^ tumour-associated macrophages (Fig. 5i–k) and neutrophils (Fig. 5l–n) in IL-33-expressing A9 tumours compared to unmodified A9 tumours. Eosinophils could be seen at the tumour periphery in metastatic A9 tumours, adjacent to the normal tissue, whereas the tumour expression of IL-33 appeared to remodel the microenvironment and allow eosinophils to flow into the tumour tissue and exert an anti-tumour effect, perhaps, due to IL-13, IL-5 production by ILC2s (Fig. 6). The increased frequency and number of innate and adaptive immune cells in IL-33 - expressing tumours supports the conclusion that tumour-infiltrating immune cells mediate protective anti-tumour immunity in murine lung carcinoma. The fact that T cells also require antigen stimulation to up-regulate expression of CD8 implies that IL-33 induction or over-expression can overcome MHC-I deficiency of metastatic tumours *in vivo.*

### IL-33 as an immune biomarker of metastatic human carcinomas

**Figure 7:**
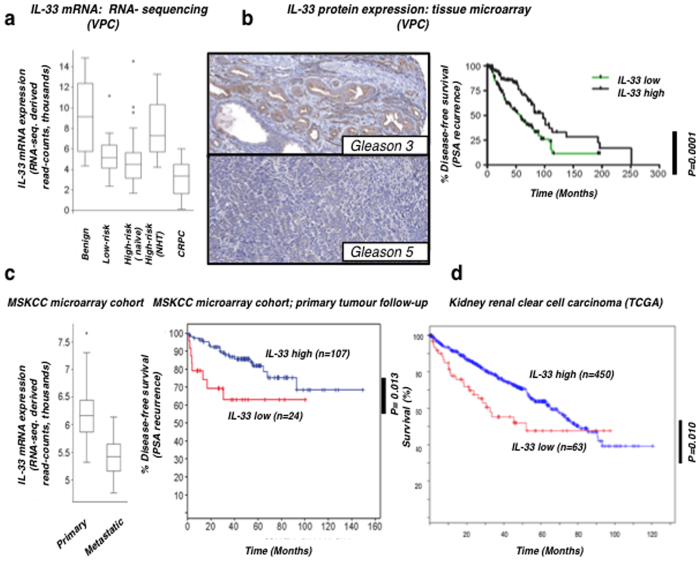
Reduced IL-33 expression is associated with prostate and kidney renal clear cell cancer progression. **(a)** Expression levels of mRNA of IL-33 in castration-resistant prostate cancer (CRPC) are low relative to benign prostate tissue and both low- and high-risk primary tumours. According to the D’Amico Risk classification: low-risk = prostate-specific antigen (PSA) less than or equal to 10, Gleason score less than or equal to 6, and clinical stage T1-2a; high-risk = PSA more than 20, Gleason score equal or larger than 8, or clinical stage T2c-3a. **(b)** Representative immunohistochemical staining (left panel) showing IL-33 expression in tissue microarrays in prostate tumours of differing Gleason grade (also known as Gleason pattern). The overall Gleason score (3 + 3) was given for the image on the top and (5 + 5) for the image on the bottom. 5μm thick sections were stained and imaged at 20x magnification. The right panel depicts the association of low IL-33 expression in primary tumours at radical prostatectomy, with significantly shorter time to PSA recurrence. **(c)** IL-33 mRNA expression in 131 case of primary prostate and 37 cases of metastatic prostate tumours confirms an association between low IL-33 expression (z-score relative to normal benign <−2) and time to PSA recurrence in this independent cohort of 131 primary prostate tumours; **(d)** association between low IL-33 expression (z-score relative to normal benign <−1) and survival of kidney renal clear cell carcinoma patients.

While we have demonstrated the importance of IL-33 in immune recognition of metastatic cancers in mice, we next tested whether this correlation held true in human clinical samples. Whereas our microarray studies indicated the expression of IL-33 may be important for cancer development, the involvement of the immune response in limiting prostate cancer and potential effectiveness of immunotherapeutic approaches in decreasing cancer recurrence is still widely debated in the literature. To begin to address this question, we examined the expression of IL-33 in prostate cancer specimens obtained at the Vancouver Prostate Centre (VPC). At the mRNA level, IL-33 expression was higher in benign prostate tissue than primary tumours that included naïve tumours (untreated prior to surgery) or NHT tumours (treated prior to surgery (p < 0.0001; t-test), and further reduced in metastatic CRPC in a large cohort of RNA-sequencing data (Fig. 7a), suggesting an inverse correlation with disease progression. Next, we evaluated the protein expression of IL-33 by immunohistochemistry in 342 prostate cancer specimens obtained at prostatectomy (Fig. 7b). Likewise, low IL-33 expression was significantly associated with reduced time to prostate-specific antigen (PSA) recurrence (a marker of relapse) after prostatectomy (p < 0.0001; Logrank test) (Fig. 7b). Patients with low tumour IL-33 expression had a median time to recurrence of 56.7 months, compared to 97 months for patients with higher tumour IL-33 expression. This effect appeared to be further correlated with Gleason grade, since there was a significant decrease in IL-33 expression within tumours of Gleason pattern 5 when compared to those of Gleason pattern 3 (Fig. 7b). To confirm this clinical association, we further explored IL-33 expression in an independent publically-available cohort of mRNA expression data from 131 cancer specimens obtained at prostatectomy including 19 metastatic tumours^45^. Again, IL-33 showed reduced expression with disease progression, and IL-33 down-regulation was significantly associated with reduced time to PSA recurrence (p = 0.013; Logrank test) (Fig. 7c). Overall this data suggests that reduced IL-33 expression in prostate cancer cells is associated with progression to metastatic disease.

We observed a similar correlation in patients with kidney renal clear cell carcinoma, using a dataset from The Cancer Genome Atlas. The report was based on analysis of 513 cases^46^. IL-33 down-regulation was significantly associated with reduced survival time (Fig. 7d). Thus, patients with low IL-33 expression had a shorter median time of survival (52.04 months), compared to patients with higher tumour IL-33 expression (80.62 months). Collectively, these data show that the reduced IL-33 expression is associated with progression to metastatic disease and reduced time to disease recurrence in patients who had resected metastatic human carcinomas.

## Discussion

In this study, we make a number of novel and potentially paradigm-shifting observations. First, we identify that IL-33 expression is reduced in many carcinomas upon their transition to the metastatic form of disease. Second, the expression of IL-33 and MHC-I by the tumours appears to be directly correlated and possibly co-regulated. Third, the re-introduction of IL-33 into metastatic tumours induces a reduction of circulating tumour cells and boosts immune recognition against metastatic tumours *in vivo*. Fourth, the expression of IL-33 may be used as an immune biomarker for recurrence and survival in human prostate and kidney renal clear cell carcinomas. Overall the down-regulation of IL-33 takes place concomitantly with the transition from primary to metastatic tumours and represents an entirely new form of tumour immune-escape.

We initially performed microarray analysis to define the metastatic gene signature network within cancer cells. While we identified many important potential regulators of immune evasion and inflammation that were significantly increased or down-regulated in both metastatic forms of prostate and lung cancers, we specifically focused on the “alarmin” IL-33 that is associated with the inflammatory process, immune recognition and functional development of the newly discovered ILC2s. This observation is the first that suggests loss of IL-33 by primary cancer cells is a component of the immune-escape pathway leading to the progression to metastatic forms, possibly, through an immune-selection mechanism akin to antigenic drift in viruses.

We identified IL-33 to be important for the immune recognition of murine lung, human kidney renal clear cell and in human and murine prostate, carcinomas. In our previous studies, it has been shown that down-regulation of MHC1-related genes ultimately leads to a reduction of immune-surveillance in metastatic cancers^4^,^5^,^38^,^41^,^59^. The present study indicates that complementation of tumour cells with IL-33 induces MHC-1 production and contributes to a reversal of antigen presentation deficiency, consequently shifting the tumour phenotype from immune evasiveness to immune recognition. The opposite effect, however, has been obtained after inhibition of IL-33 in primary tumour cells. Not only does IL-33 appear to be important for the expression of the immune-recognizable phenotype in primary tumour cell lines, but also MHC-I and IL-33 appear to be correlated during the metastatic re-programming of primary murine lung tumour revealing the metastatic potential of *IL-33* gene.

The IL-33/MHC-I correlation is a very important aspect of this study. IL-33 is a strong inducer of MHC-I and antigen processing in metastatic cancers. While the down-regulation of IL-33 ultimately decreases functionality of MHC-I and reduces immune-surveillance, the overexpression of IL-33 reverses the antigen presentation deficiency in murine metastatice lung carcinomas, and makes the tumour noticeable to both innate and adaptive immune pathways. The current paradigm in immunology declares that emergence of tumours can be limited by a robust adaptive immune response generated by the interaction of a CD8^+^ T cell with a TAA-MHC-I or a TSA-MHC-I complex. This mechanism of immune-surveillance is thought to work efficiently until tumour cells undergo chromosomal alterations that result in a loss of the expression of APM components and subsequent conversion to an immune-escape phenotype. While the most frequent immune escape mechanism is the reduced production of components from a cassette of genes involved in antigen processing and presentation^4^,^47^,^48^, downregulation of these genes ultimately decreases functionality of MHC-I, and reduces immune-surveillance. The relevance of this observations in cases of human cancer has been shown in lung^37^, melanoma^49^, renal carcinoma^50^, colorectal carcinoma^51^, head and neck squamous cell cancer^52^, cervical^53^ and breast cancers^54^ with a similar statistically significant relationship. Our study model addresses a key mechanism in immune responsiveness vs. immune evasiveness that underpins immune-surveillance to cancers. While the growth and expansion of primary tumour remains hampered by the immune system, the immune-evasive metastatic tumour phenotype allows it to acquire a growth advantage resulting in metastatic spread of the disease and its enhanced severity. Thus, the MHC-I and IL-33 co-relationship introduces a novel mechanism of cancer immune escape during tumour development, revealing the involvement of IL-33 into cancerogenesis.

Recent studies have provided equivocal results and shown both protective and promoting effects of IL-33 in tumorigenesis. The present study resolves this debate. Overall our data shows that the reduced IL-33 expression in tumours is associated with progression to metastatic disease. In addition, we demonstrate that *IL-33* down-regulation is significantly associated with reduced survival time in human prostate cancer (97 months compared to 56.7 months). We found a similar relationship in patients with kidney renal clear cell carcinoma (80.6 months versus 52 months; TCGA, access date: March, 2015). The present human studies conclusively demonstrate that the level of *IL-33* gene is downregulated in human metastatic prostate cancer compared to human benign and primary prostate tumours. The immunohistochemical staining of human prostate tumour tissue samples shows an agreement between mRNA and protein expression levels, and for the first time demonstrates a correlation to disease outcome, where low expression of IL-33 in radical prostatectomy specimens is associated with a significantly decreased time to relapse after surgery compared to specimens with high IL-33 expression. Collectively, these results indicate that the expression level of IL-33 may be a potential candidate as the first immune prognostic marker for prostate tumour transition to its metastatic form. In order to prove that, future mulitvariant analysis will be required to evaluate IL-33 mRNA/protein expression levels with clinicopathological variables which are currently used as important diagnostic features in prostate cancer, such as stage of disease, PSA level, and Gleason Score. Future studies may provide support that IL-33 down-regulation is a common thread in other carcinomas.

Only a small fraction of patients survive metastatic forms of cancer. A profound limitation in the application of general cancer treatments is the genetic and phenotypic heterogeneity in tumours between patients. Furthermore, current immunotherapy is hampered by the need to personalize treatments based on identification of tumour-specific antigens^55^,^56^, as well as on the individual tumour-specific T cell receptor expression in responding tumour-infiltrating lymphocytes. These parameters and others, such as responsiveness to PD-1, PD-L1 or CTLA4 treatment, impact therapeutic options going forward. We have demonstrated that IL-33 restores immune-responsiveness against metastatic tumours via induction of antigen processing pathways. These studies may lead to innovative and novel forms of cancer therapies for metastatic disease based on the utilization of IL-33 that could be applied as generalized cancer treatment that restores immune surveillance and combats immune-escape.

## Material and Methods

### Cell lines

#### Murine prostate cancer model

The primary PA and metastatic LMD prostate tumours were used as models of non-metastatic and metastatic prostate cancer, respectively. PA is a primary murine prostate cancer cell line derived from a 129/Sv mouse using a mouse prostate reconstitution model system that displays high expression of MHC-I. LMD is a metastatic TAP- and MHC-I deficient derivative of PA which emerged as a metastatic daughter after escaping and metastasising from the kidney capsule during serial transfer of the PA cells^57^.

#### Murine lung tumour model

The TC1 cell line is a murine lung tumour model derived from primary lung epithelial cells of C57BL/6 mice immortalized using the amphotropic retrovirus vector LXSN16 carrying human papillomavirus genes E6/E7, and subsequently transformed with pVEJB plasmid expressing the activated human c-Ha-ras oncogene. TC1 cells display high expression of TAP-1 and MHC-I. The cell line A9 was derived from the TC1 tumour cell line and display spontaneous down-regulation of MHC-I (or MHC-I) by immunoselection *in vivo* during immunization/challenge experiment^58^. All the above cell lines were grown in Dulbecco’s modified Eagle medium, supplemented with 10% heat-inactivated fetal bovine serum, 2 mM l-glutamine, 100 U/ml penicillin, 100 μg/ml streptomycin, and 10 mM HEPES.

#### Microarray analysis of both cell systems

Purified mRNA samples from both cancer cell model systems were sent to the Microarray Centre at the University Health Centre (now called The Princess Margaret Genomics Centre) in Toronto, Canada, where they were hybridized to a 28005 Two-Color Agilent microarray with a total of 55821 probes. Data analysis was performed using GeneSpring GX software (Agilent Technologies).

### Gene expression constructs and transfection

Note: untransfected tumour cells were used for microarray studies and for siRNA studies.

#### Transient clones

A gene expression construct with full-length cDNA for the selected mouse IL-33 gene (NM_001164724.1) was produced using the pIRES2-EGFP vector (Clontech Lab). Vector constructs, *i.e.* either vector alone or vector plus IL-33 gene, were transfected into both the primary TC1 cell line and the antecedent immune evasive, MHC-I deficient cell line, A9, using a FuGene 6 transfection reagent (Promega).

#### Stable clones

A gene expression construct with full-length cDNA for the selected mouse IL-33 gene (NM_001164724.1) was produced using the pIRES2-EGFP vector (Clontech Lab). TC1 and A9 cells were transfected with the pIRES2-EGFP-gene constructs using FuGene 6 transfection reagent (Promega) *in vitro.* Forty-eight hours after transfection GFP-positive cells were sorted by FACS (BD Aria II cell sorter) in order to obtain stable transfectants. Selection and expansion in culture was repeated twice before the cells were finally sorted into single-cell clones. Stably transfected clones were further isolated by flow cytometry from a population of GFP-positive cells. These stable GFP-expressing cells were used for all mouse studies.

#### siRNA study

TC1 cells were transfected with siRNAs targeted against IL-33 (GS 77125), against MHC-I (GS 1027416), untargeted siRNA (1022076) using HiPerFect Transfection reagent (301704) or left untreated. All reagents were purchased from Qiagen. The expression level of IL-33 and MHC-I was assessed in 72 to 96 hours.

#### Reverse Transcription

Total cellular RNA was extracted using Illustra RNAspin Mini Kit (GE Healthcare Life Science). Reverse transcription of 1 μg of total cellular RNA was performed using the reverse transcription kit (SSII RT) from Invitrogen.

#### Real-time quantitative PCR analysis (qRT-PCR)

Purified genomic DNA was used as a template for amplifications using 200 to 500 nM of each primer and 1 μl SYBR Green *Taq* ReadyMix (Roche) in a total 10 μl reaction mixture. Thirty-five to forty cycles of denaturation (5 s, 95 °C), annealing (5 s, 61 to 63 °C), and elongation (20 s, 72 °C) were carried out using a Roche LightCycler 480 instrument.

#### Western blots

RIPA Lysis Buffer System (sc-24948, Santa Cruz Biotechnology) was used for protein isolation from cells and tissues. To shear genomic DNA, lysed samples were passed ten times through a 21-gauge needle then incubated on ice for 30 minutes. The homogenate was centrifuged at 4 °C at 14000 × g for 10 minutes. Protein concentrations from the supernatants were determined by BCA assay (Pierce) and samples were adjusted to final concentration of 50  μg per lane. Proteins were separated with 10% sodium dodecyl sulfate-polyacrylamide gel electrophoresis and transferred to nitrocellulose membranes (Bio-Rad). Blots were blocked with 5% skim milk in phosphate-buffered saline and incubated overnight at 4 °C with the anti-IL-33 [Nessy-1] (ab54385, Abcam) mouse monoclonal antibody, mouse antiserum directed toward the region of MHC-I encoded by exon 8 (kindly provided by Dr. David Williams, University of Toronto), rabbit anti-mouse TAP-1 polyclonal antibodies (made in-house by W. Jefferies’ lab^59^), followed by the secondary antibodies, which were complimentary to the species of the primary conjugated with Alexa Fluor 680 (Invitrogen).

### Antibodies, Reagents, FACS Sorting, and Analysis

#### Antibodies used for flow cytometry to check for chimerism in mice after bone marrow transplantation

FITC-conjugated CD45.1 (11-0453-81, #A-20) and PerCP-Cy5.5-conjugated CD45.2 (45-0454-80, #104) were purchased from Affymetrix eBioscience;

#### Antibodies used to block Fc receptors

CD16/32 (564220, BD Pharmingen).

#### Excluding nonviable cells from flow cytometry

Fixable Viability Dye eFluor 780 (65-0865-14, Affymetrix eBioscience).

#### CD4, CD8 staining

APC-conjugated antibody against CD4 (553051, RM4-5, BD Bioscience) and PE-Cy7-conjugated antibody against CD8a (25-0081-82, 53-6.7, Affymetrix eBioscience).

#### MHC-I expression

PE-conjugated anti-Kb mouse monoclonal antibody (553570, BD Pharmingen).

#### Flow Cytometry

BD FACS Aria II was used for cell sorting and phenotypic analysis. The program FlowJo version10.1 was used for data analysis.

#### Mice

C57BL/6 mice were purchased from the Jackson Laboratories and maintained in the British Columbia Cancer Research Centre (BCCRC) pathogen-free animal facility. Mice were used at 4–8 weeks of age. These experiments were approved by the Animal Care Committee of the University of British Columbia. Animals were maintained and euthanized under humane conditions in accordance with the guidelines of the Canadian Council on Animal Care.

#### Tumour establishment

Genetically modified tumour cells (50 μl of 5 × 10^5^) were injected into chimera animals subcutaneously into the right flank. Tumour growth was monitored by measuring tumour dimensions with calipers. Tumour length and width measurements were obtained three times weekly. Tumour volumes were calculated according to the equation tumour volume=length x width x height x π/6 with the length (mm) being the longer axis of the tumour. Animals were weighed at the time of tumour measurement.

#### Primary Leukocyte Preparation

Cell suspensions were prepared from tumours and lymph nodes^60^. Tissues were cut into small pieces with a razor and digested for 40 min in MEM, 10% FBS, penicillin and streptomycin (P + S), 50 mM 2-mercaptoethanol, Collagenase IV (Invitrogen), and DNase (Sigma) at 37 °C. Digested tissue was pushed through a 70 μm strainer, and Percoll (GE Healthcare) gradient enrichment of leukocytes followed.

#### Immunohistochemistry

Tumours were embedded in Tissue-Tek O.C.T. media (Sakura) on dry ice and immediately stored at −80 °C until sectioning. 10 μm thick sections were collected on Leica cryostat and stored at −80 °C until staining. Slides were removed from −80 °C, fixed in cold acetone or acetone:methanol. Following washing in PBS, slides were incubated with protein block and subsequently incubated with specific antibodies overnight. Antibodies used: anti-CD4 (553043; BD Bioscience), anti-CD8 (553027; BD Bioscience), anti-MHCI (15681; Abcam), anti-FoxP3 (54501; Abcam), anti- Ly-6G (MAB1037; R&D System), anti-CD68 (53444; Abcam). Appropriate horseradish peroxidase (HRP) conjugated secondary antibodies were used for detection of the primaries and developed with DAB chromogen. Purified Rat IgG2a, k for the CD8a and CD4 (553027; BD Pharm); clone RTK2758 for the MHCI (70636; BioLegend); for Ly6G (400601; Biolegend); for FoxP3 (I-1000; Vector) were used as negative controls. Slides were counter stained with haematoxylin and eosin (H&E) and dehydrated in ethanol and xylene. Slides were then cover slipped and imaged with an Aperio ScanScope at 20x magnification.

### Human Prostate Samples

#### mRNA Sequencing Cohort

RNA-sequencing data from the Vancouver Prostate Centre was obtained and analyzed exactly as previously described^61^. Public data from Taylor *et al*.^45^ was explored using the cBioPortal^62^.

### Immunohistochemistry

Immunohistochemical stains were conducted at the Vancouver Prostate Centre using a Ventana autostainer model Discover XT (Ventana Medical System) with enzyme labeled biotin streptavidin system and solvent resistant DAB Map kit using 1/50 concentration of rabbit polyclonal IL-33 antibody (HPA024426; Sigma-Aldrich). Staining was performed on 342 prostate cancer specimens obtained from the Vancouver Prostate Centre. The H&E stained slides were reviewed and the desired areas were marked on them and their correspondent paraffin blocks. Five tissue microarrays (TMAs) were manually constructed (Beecher Instruments) by punching duplicate cores of 1mm for each sample. Stained slides were digitalized with the SL801 autoloader and Leica SCN400 scanning system (Leica Microsystems) at magnification equivalent to ×20. Representative cores (clearly positive, clearly negative and mixed positive/negative) were manually identified by an experienced pathologist (L Fazli) and a four-point scale was assigned as follows: 0 represents no staining in any tumour cells, 1 represents a faint or focal, or questionably present stain, 2 and 3 represents a stain of convincing intensity in a majority of cells. For comparisons, a score of 0 or 1 was considered low IL-33 expression.

#### Statistics

Data were analyzed with Excel. A Student’s t test was used for determining statistical significance between groups; p ≤ 0.05 was considered significant. The statistical analysis of microarray results was carried out with FlexArray (Genome Quebec).

## Additional Information

**How to cite this article**: Saranchova, I. *et al*. Discovery of a Metastatic Immune Escape Mechanism Initiated by the Loss of Expression of the Tumour Biomarker Interleukin-33. *Sci. Rep.* **6**, 30555; doi: 10.1038/srep30555 (2016).

## Supplementary Material

Supplementary Information

## Figures and Tables

**Table 1:** IL-33 gene-complementation inhibits metastatic spread of tumour cells to distal organs.

Tumour Cell	Control (no tumour cells)	A9	A9+IL-33	TC1
Distal Organ				
Liver	0.00%	24.0–32.3%	0.0–0.2%	0.00%
Adrenal glands	0.00%	3.7–14.9%	0.9–4.3%	0.00%
Lungs	0.00%	0.00–1.3%	0.00–0.2%	0.00%
Blood	0.00%	Single cells 1–33 cells/0.5 ml = 0.00%	0.00–0.2%	0.00%
Brain	0.00%	0.00%	0.00%	0.00%

GFP-positive circulating tumour cells were isolated from sites that were distal from initial subcutaneous inoculation and assessed using flow cytometry. Shown here are representative results from each of the four tumour groups, where n = eight animals/group.
