# Supplementary material for: Discovery of a Metastatic Immune Escape Mechanism Initiated by the Loss of Expression of the Tumour Biomarker Interleukin-33
Source: Sci Rep. 2016 Sep 13;6:30555. doi: 10.1038/srep30555 (PMC5020406; doi:10.1038/srep30555)
Supplement: Supplementary Information [file srep30555-s1.pdf]

Supplementary Information:

Title: Cancer Immune Escape in the Primary to Metastatic Transition is Guided by the Loss of Interleukin-33 Expression

Authors: Iryna Saranchova, Hui Huang, Franz Fenninger, Kyung Bok Choi, Lonna Munro, Cheryl Pfeifer, Ian Welch, Alexander W. Wyatt, Ladan Fazli, Martin E. Gleave, Wilfred A. Jefferies*

Tissue or Organ \ Tumour Type	A9	A9+IL33	TC1
**Tumour CD8**	1.45%	7.01%	22.32%
**Adrenal Gland CTC**	12.32%	2.40%	0.001%
**Adrenal Gland CTC/ Tumour CD8**	8.49	0.34	0.000040
**Lymph Node CD8**	1.26%	16.1%	16.1%
**Lymph Node CTC**	4.98%	1.69%	0.001%
**Lymph Node CTC/ Lymph Node CD8**	3.95	0.10	0.000062

Table S1: Indirect correlation between the number of circulating tumour cells and the presence of CD8+ T cells. The percentage of circulating tumour cells (CTC) that can be detected in distal organs is significantly lower in tumours which express IL-33. Reduced CTCs also correspond to increased percentages of CD8+ cytotoxic T cells found in both the tumour and in local lymph nodes. Percentages of cells are determined as a percentage of all cells in the tissue/organ. CTCs were assessed as GFP-positive cells.
